# NKT cells in the antitumor response: the **β** version?

**DOI:** 10.1172/JCI177663

**Published:** 2024-02-15

**Authors:** Mitchell Kronenberg, Isaac Engel

**Affiliations:** Center for Infectious Disease and Center for Autoimmunity and Inflammation, La Jolla Institute for Immunology, La Jolla, California, USA.

## Abstract

NKT cells recognize glycolipids presented by CD1d-expressing antigen-presenting cells (APCs) and include type I NKT cells with antitumor function and type II NKT cells, which have been reported to suppress the antitumor response. Some type II NKT cells recognize sulfatide, a glycosphingolipid with a sulfate modification of the sugar. Type I NKT cells recognize different glycosphingolipids. In this issue of the *JCI*, Nishio and colleagues showed that APCs could process sulfatide antigens, analogous to protein processing for peptide-reactive T cells. Antigen processing in lysosomes removed sulfate to generate a glycosphingolipid that stimulated type I NKT cells and thereby turned an antigen with no antitumor activity into one that not only stimulated type I NKT cells but also stimulated antitumor responses. These findings may extend to the development of glycolipid antigens that could stimulate anticancer responses via antigen processing by APCs.

## Type I and type II NKT cells recognize different glycolipids

**Figure 1:**
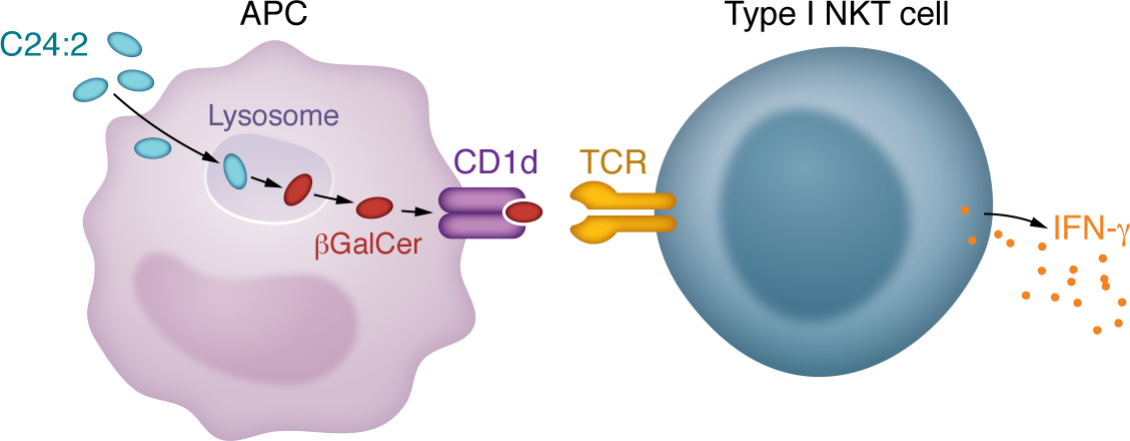
APCs stimulate type I NKT cells and the antitumor response via lysosomal generation of a modified glycosphingolipid. APCs exposed to C24:2 generate βGalCer via lysosomal processing, which removes a sulfate. NKT cells recognize glycolipids presented by CD1d-expressing APCs. Notably, βGalCer stimulates type I NKT cells to have an antitumor response through the production of IFN-γ.

NKT cells, which are T lymphocytes that are distinct from innate immune NK cells, have been tested extensively for anticancer responses on the basis of the initial positive findings that they prevent cancer metastases in mice (1). NKT cells are defined generally as T lymphocytes that recognize glycolipid antigens presented by CD1d, a nonpolymorphic antigen-presenting molecule (2). Because CD1d is not polymorphic, agents that stimulate NKT cells should activate these lymphocytes in all individuals, and not be limited by the vast polymorphisms in HLA antigen-presenting molecules that define the responses of other T cells. In this issue of the *JCI*, Nishio and colleagues define a cellular mechanism in DCs that converts a synthetic glycosphingolipid antigen into a more effective anticancer agent (3) (Figure 1).

Structural studies of the NKT cell antigen receptor (TCR) in a complex with CD1d and antigen reveal a shape in which the lipid chains are buried within the hydrophobic CD1d antigen–binding groove (4). The sugar sits at the surface of the glycolipid-CD1d complex, where it provides contact points with the NKT TCR (4). A complicating factor in analyzing the anticancer response of NKT cells, however, is that there are two categories, and only one of them, so-called type I NKT cells, has anticancer activity in mouse studies. The other NKT cell subset, type II NKT cells, is reported to be suppressive of the tumor response (5). Type I NKT cells express a limited TCR repertoire with an invariant TCRα chain (6). These cells recognize several types of glycolipids, mainly glycosphingolipids (GSLs) that are composed of a ceramide lipid with an α stereochemical linkage of the sugar to the lipid. This α-linked structure is found in some types of bacteria (7, 8), while in contrast, most mammalian GSLs have a different stereochemistry, a β-linked sugar. GSL antigens with β-linked sugars minimally activate type I NKT cells, but can still activate them (9, 10). Notably, the structural data indicate that the α-linkage of the sugar in GSL antigens is critical for the optimal fit with the type I NKT cell TCR containing an invariant α chain (4). Because strong antigen activation of mouse type I NKT cells by GSLs with α-linked sugars showed anticancer efficacy in mice, and these compounds activate human type I NKT cells (11), cancer clinical trials were initiated (12), and many trials followed. Although antitumor efficacy was limited in patients (13), attempts are underway to synthesize more effective GSLs for cancer treatment and as vaccine adjuvants for infectious diseases (14).

Type II NKT cells exhibit a more diverse TCR repertoire than do type I NKT cells. Although studies suggest type II NKT cells can recognize different types of glycolipids (15), recognition of sulfatide, a GSL with a sulfated β-linked galactose sugar, has been reported to activate at least some type II NKT cells (16). Studies of NKT cell sulfatide reactivity have used natural sulfatides, which have the same carbohydrate moiety but complex mixtures of ceramide lipids. This natural heterogeneity could be important for influencing the immune response, however, because subtle changes in the ceramide lipid structure, such as the addition of one or more double bonds, or alteration of the hydrocarbon chain length, have been shown to lead to dramatic changes in the NKT cell immune response (17). Therefore, a key feature of the Nishio et al. study was the use of synthetic sulfatide antigens with defined ceramide fatty acid chains, either with zero, one, or two double bonds, and immune assays using well-characterized immortalized mouse type I and type II NKT cell hybridomas.

## Lipid structure affects NKT cell activation

The authors analyzed the reactivity of a type II NKT cell hybridoma and several type I NKT cell hybridomas to synthetic sulfatides in culture wells coated with soluble CD1d protein. Therefore, they could directly assess TCR reactivity in the absence of costimulatory or other signals from the antigen-presenting cells (APCs), and they confirmed that the structure of the lipid influenced TCR-mediated activation. Importantly, sulfatide C24:1, with a single unsaturated bond in the fatty acid, and C24:2, with two unsaturated bonds, activated type II but not the type I NKT cell hybridomas. The results were reversed, however, when the NKT cell hybridomas were stimulated with the synthetic sulfatide antigens that were cultured with bone marrow–derived DCs that expressed CD1d. In this case, only type I, not type II, NKT cells were stimulated. The stimulation of type I NKT cells was more effective with C24:2 than C24:1, although the structural basis for this remains unclear. These data suggest that the DCs altered or processed the sulfatide to change the NKT cell type they stimulated.

## Lysosomal antigen processing stimulates type I NKT cells

CD1d is most often loaded with stimulatory antigens in lysosomal compartments. In lysosomes, aryl sulfatase A can cleave the sulfate from sulfatides to generate a GSL with a β-linked sugar, β-galactosyl ceramide (βGalCer). Nishio and colleagues showed that βGalCer, the presumptive product of aryl sulfatase A–mediated antigen processing, stimulated the type I, but not the type II, NKT cell hybridomas when it was cultured on CD1d-coated plates, although it was less stimulatory than a synthetic αGalCer counterpart. Additional experiments showed that either the inhibition of endosomal acidification, the inhibition of the aryl sulfatase A enzyme with sulfite, or stimulation with APCs deficient for the enzyme all abolished the ability of the synthetic sulfatide to stimulate type I NKT cell hybridomas. The response to βGalCer was not affected, ruling out nonspecific effects. Presumably, the lack of aryl sulfatase A activity would have allowed the DCs to stimulate the type II NKT cell hybridomas in the presence of C24:2, although this was not shown. Regardless, overall, the data are consistent with a model in which glycolipid antigen processing, meaning sulfate cleavage from the sulfatide GSL in lysosomes, changes antigen structure from sulfatide GSL to βGalCer and, consequently, antigen reactivity from type II to type I NKT cells.

## Sulfatide antigen processing stimulates the anticancer response

The reductionist approach undertaken by Nishio and colleagues has the weakness that only a few mouse T cell hybridomas were analyzed, but the authors followed up by showing that human type I NKT cells could be activated in vitro by C24:2. Antigen stimulation was decreased by inhibiting endosomal acidification, suggesting that stimulation of human type I NKT cells by C24:2 required antigen processing. Furthermore, in vivo experiments showed that C24:2 provided protection from metastases of the CT26 colon cancer to the lung, although C24:1, less stimulatory for type I NKT cells, did not have an effect on tumor nodules. The tumor protection by C24:2 injection was dependent on IFN-γ synthesis. Spleen and lung mononuclear cells from mice injected with C24:2 produced more cytokines, especially IFN-γ, than did those from mice injected with C24:1, and cytokine secretion was dependent on CD1d expression. Activation by C24:2 led to an increased number of type 1 conventional DCs (cDC1s), which are important for tumor immunity and production of IL-12. These are all features earlier shown to be important for the antitumor response by type I NKT cells (18). Mice deficient for *Traj18*, the single TCR Jα segment required for type I NKT cells, express CD1d and therefore also retain type II NKT cells in the absence of type I NKT cells. C24:2 or βGalCer injection did not reduce tumor metastases in these mice, confirming the requirement for processed C24:2 to induce the anticancer response by type I NKT cells.

Although the processing of glycolipid antigens by APCs had been defined previously in a few studies (19), the work by Nishio et al. definitively shows that antigen processing can affect the type of glycolipid-reactive T cell that is stimulated, with corresponding effects on the antitumor response. C24:2 and βGalCer are not particularly strong stimulators of type I NKT cells, however, and are therefore unlikely to be important in unmodified form for human cancer immunotherapy. Still, the findings may lead to the design of more effective glycolipid antigens that DCs might process to provide an anticancer therapy through NKT cell stimulation.

## Version 1 02/15/2024

Electronic publication
